# Gender and Childhood Victimization: A Longitudinal Study of Heavy Drinking in Young Adulthood

**DOI:** 10.3390/ijerph182111089

**Published:** 2021-10-21

**Authors:** William Ash-Houchen, Celia C. Lo, Heather M. Gerling, Tyrone C. Cheng

**Affiliations:** 1 Department of Justice Studies, Prairie View A&M University, Prairie View, TX 77446, USA; 2 PERSEREC, Peraton, Seaside, CA 93955, USA; celiaclo@yahoo.com; 3 Center for Depression Research and Clinical Care, UT Southwestern Medical Center, Dallas, TX 75390, USA; heather.gerling@utsouthwestern.edu; 4 School of Social Work, University of Alabama, Tuscaloosa, AL 35401, USA; tyronecheng@yahoo.com

**Keywords:** heavy drinking, victimization in childhood, depression, longitudinal study, gender

## Abstract

The present longitudinal study, for 12 years, followed a group of young adults, examining (1) whether/how victimization in childhood increased the likelihood of heavy drinking; (2) whether depression mediated the strain–heavy drinking relationship; and (3) whether/how relationships among strain, depression, and heavy drinking differed across two gender groups. Data came from the National Longitudinal Survey of Youth 1997 cohort, dating 2004–2015 (5 interview waves and 22,549 person-wave measurements total). We linked consumption of 5+ drinks (during the month prior) to four discrete measures of violent victimization, to one measure of stressful events, and to depression. We needed to consider repeat measures of the same variables over time, so we used generalized estimating equations (GEE) to analyze data. Depression was found to increase heavy drinking uniformly. Empirical evidence confirmed that in the strain–heavy drinking relationship, depression plays a minor mediating role. Gender moderated heavy drinking’s associations. Specifically, bullying in childhood raised risk for female respondents. The current strain was associated with a higher risk of heavy drinking among male respondents. Childhood victimization, as well as current life stress, play an important role in depression and heavy drinking. Future research should focus on the development of specific, targeted care to reduce heavy drinking’s harm and promote equity among Americans.

## 1. Introduction

Most often defined as four or more drinks (for women) and five or more drinks (for men) in a relatively short window of time [1], heavy drinking remains a relatively common behavior among older adolescents and young adults, often tapering off after the transition to adulthood [2,3]. Close links are found in the literature between substance use and mental health symptomatology, such as depression, with some cases identifying substance use as a cause and others classifying substance use as an effect [4,5]. Heavy drinking during adolescence is associated with an array of harmful outcomes, from biological changes such as reductions in gray brain matter in the brain [6], greater risk of alcohol use disorder in adulthood [7], aggressive behavior and fighting [8,9], dating violence [10], reductions in health-related quality of life [11], other substance use [12], and socio-legal consequences (e.g., arrest) [13,14] and is thusly examined from public health and criminological contexts.

Recent research into heavy drinking behavior has elucidated nuances related to gender and race and still other statuses, often operating in tandem, that further refines our understanding of the complex interplay of social structure and lived experience. Heavy drinking occurs among all racial groups, with a general understanding that it is more common among male and White adolescents and young adults [15,16]. There is a significant body of research, however, examining gender differences in heavy drinking, finding that some statistical relationships (e.g., heavy drinking and fighting) act stronger for female respondents than males, making outcomes among female respondents worse [17,18]. Relatedly, while adolescents may be exposed to or model parental drinking behavior, parental heavy drinking may more powerfully impact young girls vs. boys in adolescence [19]. A significant and lasting relationship between victimization and substance use has been elucidated in the literature [20,21,22,23] explained using theoretical perspectives from public health and criminological disciplines, including the self-medication hypothesis, general strain theory, and the stress-process model. The effects of victimization, however, do not exist solely for those who are direct victims, such as in the case of a bullied child or adolescent. Instead, vicarious victimization, such as witnessing another person being shot or otherwise indirectly encountering victimization as a bystander, has become an increasingly powerful predictor of future substance use and depression in need of further examination [24,25,26,27].

### 1.1. Theoretical Frameworks

Three theoretical frameworks, the self-medication hypothesis, general strain theory, and the stress-process model, each posit overlapping elements of a stress and substance use coping relationship. Each shares a central tenet that stressful events in life can affect levels of substance use, although pathways differ among each. Rather than being seen as entirely competing perspectives on explaining heavy drinking behavior among adolescents, instead, these perspectives together help frame relevant predictors of adolescent heavy drinking, such as objective (e.g., poverty) and subjective stress/strain (e.g., victimization), mental health symptomatology, and a presence or lack of social support.

Khantzian’s [28,29] self-medication hypothesis posits disruptions in affect and the ability to manage one’s affect can induce a desire to reduce or eliminate such pain with substance use of a type that would diminish the experienced symptoms. If an individual were experiencing high levels of anxiety, they might then turn to depressant substances to help manage those symptoms. The self-medication hypothesis’ associations with substance use have been tested in a variety of contexts in the historical literature [30,31,32]. Mentions of self-medication are now rarer, with some calls to retire the framework entirely in discussions of psychiatric conditions [33]. What remains trenchant from self-medication is the idea that painful affect can induce substance use, including heavy drinking [34].

Agnew’s general strain theory [35,36,37] was developed to better explain violent delinquency, suggesting that the removal of positive stimuli, the introduction of negative stimuli, or a failure to achieve goals can each operate on a mediating negative affective state (often anger), and this negative affect can precipitate coping through criminal means such as substance use or delinquent behavior. General strain theory also confirms stressors’ significance over time since stressors foster emotional or affective states (depression, anger, etc.) associated with deviance, including substance use [20,38]. For example, empirical research has found that use/misuse of alcohol can function as a coping strategy among children, adolescents, and adults exposed to stress from a variety of sources [39,40,41].

Pearlin’s [42,43] stress-process model understands stressful events as a part of life, either acute or chronic, but often structured by race, gender, socioeconomic status, and still other forms of stratification leading to unequal distributions within and across communities. Social support, either from social networks or intimate partners, provides a critical cushion against these strains. Per the stress-process model, encountering stressors is a predictor of diminished mental health; at times, individuals respond to diminished mental health with drug use that constitutes self-medication [44].

### 1.2. Victimization, Depression, and Heavy Drinking

Victimization’s relationship to increased risk of heavy drinking is documented in the literature [9,45,46], most often understood in the broader context of adverse childhood experiences (ACEs) and negative associations with mental health, across the lifespan, for both women and men [47,48,49,50,51,52,53]. Verbal, physical, and sexual abuse, as well as exposure to violence within the household, are all elements of adverse childhood experiences. While generally understood in the literature to increase the risk of engaging in heavy drinking, that victimization’s effects are uniform for racial and ethnic minority groups or for women versus men cannot be taken for granted [52,53]. As a codifying example, sexual assault histories are widely reported among women, and ample research indicates these histories matter both as relevant risk factors but also avenues for heavy drinking treatment addressing underlying traumatic experiences [54,55]. Critically, coping with victimization may present further opportunities for additional or repeated victimization. Coping with the distress of an assault may lead one heavy drinking behaviors, in turn raising the risk of re-victimization [56].

Rich empirical evidence associates stressful and straining events with heavy drinking as an outcome in the short- and long-term [5,8,9,45,46,53,55,57,58]. Psychological distress, often assessed as depression, is a common predictor of heavy drinking [5,13,18,59], and the same stimuli driving levels of depression may also change the risk and protective factors for substance use behaviors [44]. Factors such as poverty and unemployment are related to heavy drinking throughout the literature, generally finding uniform effects leading toward higher levels of heavy drinking when unemployment and poverty levels are high [16,58,60]. That these periods are also associated with higher levels of depression is expected [61]. Higher levels of social support, often through an intimate partner, exert a protective effect against heavy drinking [16,55]. Protective resources may also exert differential and more powerful effects for women vs. men [62]. Previous research has also highlighted the positive effects of treatment, often brief motivational interviewing or interventions, with these efforts retaining the capacity for tailoring to specific audiences [17,18,55,63,64].

### 1.3. Hypotheses

The present study aimed at identifying a social mechanism that might connect childhood victimization to adulthood heavy drinking and depression using a framework including elements of the self-medication hypothesis, general strain theory, and the stress-process model. These perspectives suggest a general trajectory of higher risk of engaging in substance use, including heavy drinking, in the presence of current or former stressful events or strain, mental health symptomatology, and a lack of social support. Our prospective longitudinal design and multiple measures of respondents’ heavy drinking as they aged allowed the study to track the impact of childhood victimization across a decade. The data collected in 2004, 2006, 2008, 2010, and 2015 described respondents as they reached ages anywhere from 19 to 36. That data meant we could take time into consideration as we observed what happened to effects of childhood victimization as adulthood progressed (e.g., their potential diminishment). We hypothesized (1) *direct* forms of childhood victimization, being bullied, and one *vicarious* form of childhood victimization, witnessing the shooting of a person, functioned as a mechanism linking victimization and adult heavy drinking and depression. (2) We further hypothesized depression mediates any relationship between childhood victimization and heavy drinking as is outlined in the criminological and mental health frameworks of stress-process, self-medication, and general strain. (3) Because we posited that a victim’s age when victimized might be associated with differential impacts of victimization, we measured both of our childhood victimization variables twice: when respondents were preadolescents age 11 or younger and when they were 12–18. Moreover, we assigned a time factor to childhood victimization, namely, before or after age 12 (but no later than age 18). (4) We could find limited empirical evidence of gender’s possible association with heavy drinking among individuals who experienced direct or vicarious victimization during preadolescence or adolescence [21,65] warranting further examination.

## 2. Materials and Methods

### 2.1. Data and Sample

The National Longitudinal Survey of Youth 1997 (NLSY97), funded by the Bureau of Labor Statistics, provided data for the present longitudinal study. The Ohio State University had begun the survey as an annual data collection, making it biennial after 2011. The survey sought to capture individuals’ life experiences, notably, those involving family, crime, health, and substance abuse, over time. Its national representative sample comprised civilian, non-institutionalized people born between 1980 and 1984, who responded to items posed by an interviewer using computer-assisted personal interview (CAPI) technology, either in person or by phone. Respondents chose to be interviewed in English or in Spanish. A supplemental survey oversampling Black and Hispanic/Latino youth (born 1980–1984) was also included in the full sample of NLSY97. In 1997, the researchers interviewed 8984 persons age 12–18. Other descriptions of NLSY97 are included in previous research [41,66].

### 2.2. Measures

Our outcome variable, heavy drinking in the past month, was measured during 5 NLSY97 survey years between 2004 and 2015 (inclusive). We created for each respondent a longitudinal record that linked data from the 5 survey years. From each such record, we derived a number of person-waves, which provided our units of analysis; we applied the discrete-time method to analyze the data [67]. Within our final sample were 11,210 person-waves describing male respondents and 11,339 person-waves describing female respondents, for a total of 22,549 person-waves.

We measured the time-varying outcome variable, heavy drinking in the past month, for the NLSY97 survey years 2004, 2006, 2008, 2010, and 2015. Data from only these years made up the longitudinal records in our final sample, via which we measured respondent likelihood of recent heavy drinking. The measure reflected NLSY97 respondents’ reports of how often (i.e., on how many days), in the 30 days preceding interview, they had consumed 5 or more drinks. A 1, indicating heavy drinking, was assigned to a respondent reporting consumption of 5 or more drinks on at least 1 day of the 30; the 0 assigned to respondents not reporting such consumption constituted the reference.

We employed 12 independent variables: gender, race/ethnicity, depression, recent stressful events, income-to-poverty ratio, education, age, marital status, parent’s education, bullying victimization before age 12, bullying victimization at age 12–18, seeing the shooting of a person before age 12, and seeing the shooting of a person at age 12–18. Level of depression, income-to-poverty ratio, education, age, and marital status were treated as time-varying variables; all others were considered time-invariant. Depression was measured with a 5-item index asking how often, in the 30 days preceding survey, a respondent had felt nervous; calm or peaceful; down or blue; happy; and depressed. The index’s offered responses ranged from 1 (all of the time) to 4 (none of the time); reversed coding allowed higher index scores to indicate higher levels of depression. The index’s overall reliability is indicated by alpha scores ranging from 0.78–0.81 across the 5 survey years.

We used an index of 7 types of recent stressful events to describe respondents’ experience over the previous 5 years of data collection in 2002, 2007, and 2013. The 7 included death of a close relative; becoming the victim of a violent crime; hospitalization of a member of one’s household; incarceration of a member of one’s household; a household member’s becoming unemployed; one’s parents divorcing; and one’s family becoming homeless. Index scores ranged from 0 to 7; a relatively high number indicated the experience of relatively numerous stressful events.

Concerning education, the variable high school completion indicated possession of a high school diploma or GED, and the variable college completion indicated possession of an associate or baccalaureate degree; receipt of neither diploma nor degree provided the reference. At initial interviews conducted in 1997, NLSY97 respondents were ages 12–18. We used respondent age in 1997 to construct our variable age, which we measured in each of the 5 selected survey years. For each respondent, we recorded a continuous measure of household income-to-poverty ratio for the 12 months preceding each of the 5 interviews. For each, we measured marital status dichotomously, a 1 indicating being married, 0 otherwise.

We treated as time-invariant and measured dichotomously the variables bullying victimization before age 12, bullying victimization age 12–18, seeing the shooting of a person before age 12, and seeing the shooting of a person age 12–18. We assigned a 1 to indicate the presence of any of these forms of victimization, a 0 to indicate absence (the reference). We measured gender dichotomously, 1 indicating male, 0 female. We employed two dichotomous race/ethnicity measures, with 1 indicating a respondent self-reporting Hispanic ethnicity or non-Hispanic Black ethnicity and 0 indicating self-report of non-Hispanic White ethnicity. Parent’s education was a time-invariant continuous variable stating the highest level of education attained by parents or, where mother’s level differed from father’s by that parent having the most formal education. Offered responses for this measure ranged from 0 (no education) to 7 (graduate or professional degree). Additionally, we employed dichotomous time factors describing each interview wave represented in our data: 2006, 2008, 2010, and 2015 (the 2004 survey year provided the reference).

### 2.3. Data Analysis

In light of our use of repeated measurements, we employed generalized estimating equations (GEE) to analyze the longitudinal data, estimating autocorrelations and autoregressive correlations using STATA (StataCorp, College Station, TX, USA) [68]. The analysis assessed, by gender, potential associations between the likelihood of heavy drinking and all independent variables. For the sample in its entirety, as well as for male respondents and female respondents separately, we completed a two-step multivariate analysis. For each of the three groups, Model 1 regressed heavy drinking on all independent variables except depression. We added depression to Model 1 to produce Model 2, the final multivariate model. In addition, we used the following procedure to examine gender’s possible moderating role in heavy drinking’s associations. First, we created a group of interaction terms between males and each of the other independent factors. Second, we ran a series of multivariate analyses that employed the entire sample and included (a) all independent variables, including the time factors and the dummy variable gender, and (b) interaction terms created between gender and each independent variable. Third, we tested for statistical significance to determine whether a given independent variable’s association with heavy drinking likelihood differed for male versus female respondents, with all other independent variables controlled. If no coefficient for each of the two groups proved statistically significant, a moderating effect would not be sought.

## 3. Results

Concerning our heavy drinking outcome, data from 6303 respondents (3187 males, 3116 females) drove the results of the final multivariate analysis. Of the 6303, self-reported White respondents constituted 51.4%, self-reported Black respondents were 25.3%, and self-reported Hispanic respondents were 20.6%. For the sample as a whole, bullying victimization was more pervasive than the vicarious victimization of seeing a shooting: While 20.1% reported being bullied before age 12 and 15.2% reported being bullied at 12–18 years of age, just 10.5% reported seeing a shooting before age 12, and 9.7% reported seeing a shooting at between 12 and 18 years of age. Compared to females, males in our study were significantly more likely to have been subjected to bullying victimization before age 12, as well as to seeing a shooting both before age 12 and between the ages of 12 and 18.

Table 1 shows descriptive statistics for the time-varying heavy drinking outcome and independent variables. Statistics are presented both by gender and for the sample as a whole. Using chi-square and *t*-tests, we identified significant differences across gender groups for, respectively, the categorical and continuous variables. Across the 5 survey years, we considered, 44.4% of male respondents and 25.6% of female respondents reported consuming at least 5 drinks on at least one day in the preceding 30 days. For each independent variable except age and survey year, we observed statistically significant male–female differences.

Table 2 reflects our GEE model explaining heavy drinking likelihood for the full sample. Model 1 generated statistically significant associations between heavy drinking and seeing a shooting at 12–18 years of age. Moreover, Model 1 indicated that the likelihood of heavy drinking was higher for White respondents (versus Black or Hispanic), males, holders of an associate’s or baccalaureate degree, high-income individuals, unmarried individuals, and individuals with a relatively well-educated parent(s). In turn, Model 2, which added to Model 1 the variable depression, yielded nearly identical results to Model 1. However, in Model 2, the relationship between seeing a shooting at 12–18 and heavy drinking in young adulthood did not achieve statistical significance. Nevertheless, in general, including depression did not substantially reduce the strength of the Model 1 coefficients. In this study, higher levels of depression were associated with a greater likelihood of engaging in heavy drinking in the 30 days preceding the interview. Models 1 and 2 alike exhibited significant improvement over the null model.

Table 3 presents multivariate results for male respondents and for female respondents separately. Again, our analysis included running Model 1 and then Model 2 discretely for each gender. In Model 1, male respondents were significantly less likely to report heavy drinking if they had experienced bullying before age 12, if they were Hispanic or Black if they were relatively low-income, if they reported relatively little stress, and if they were married. In Model 2, the variables that proved significant in Model 1 again proved significant, and a significant association was also found between depression and heavy drinking. In Model 2, moreover, coefficients’ Model-1 strength generally persisted, as we have said, except that bullying victimization before age 12 showed a weaker significant association to heavy drinking once depression was included in the analysis.

Female respondents in our study were significantly more likely to report heavy drinking if they experienced bullying victimization before age 12; if they were White (rather than Black); if they held an associate’s or baccalaureate degree (versus lacked a high school diploma); if their income was relatively high; if they were unmarried; or if they had a relatively well-educated parent(s). Model 2 indicated that for the females in our sample, reporting a relatively high level of depression was significantly associated with heavy drinking. Comparing Model 1 to Model 2, most coefficients did not exhibit substantial change; an exception was a slight change for the variable bullying victimization before 12. We obtained significant Wald chi-squares for all four of our models, signifying that the four represented significant improvements over the null models in the explanation of heavy drinking.

Using the procedure detailed under “Data Analysis” above, we evaluated gender’s moderating role in heavy drinking’s associations with the included independent variables. Five significant gender differences demonstrate that gender moderated heavy drinking’s associations. First, bullying victimization before age 12 reduced heavy drinking among males in our study, but it increased heavy drinking among females, according to our data analysis results. Second, Black respondents’ relatively lower likelihood of heavy drinking, compared to Whites, was more pronounced among females compared to males. Third, in comparison with White respondents, we found Hispanic males to be at lower risk of heavy drinking versus White males, but heavy drinking risk for Hispanic females was about the same as that for White females. Fourth, among all females in our study, having an associate’s or baccalaureate degree was associated with a relatively high likelihood of heavy drinking, to a degree surpassing the association found for males in the study. Finally, our study found heavy drinking to be much less likely among married respondents of either gender versus unmarried respondents of either gender, and this marriage–heavy drinking relationship was much more pronounced.

## 4. Discussion

Plainly, heavy drinking is a risky health behavior associated with many negative outcomes across the lifespan ranging from structural changes in the brain to criminal-legal interventions in adolescence and adulthood [16,45,69]. Our study examined multiple important features of heavy drinking, including its associations with powerful stressful events from childhood to adulthood and how gender modifies these statistical relationships. Findings can be interpreted as follows:

First, we acknowledge the outsized role played by victimization in patterning heavy drinking outcomes. Our study measured both direct and indirect victimization occurring in childhood and in adolescence, a total of four possible victimization measures early in the lifespan and their effects over time. This is an improvement to cross-sectional literature with more static measurements of victimization. With the control of more proximate traumatic and stressful events as indicated theoretically [37,70], the relationships among victimization and coping through heavy drinking as we hypothesized are made clearer. Our findings suggest that direct victimization in childhood is a critical predictor of later heavy drinking behavior for only female adolescents and young adults, and the same risk-generating effect was not found for our measure of vicarious or indirect victimization as expected for either gender group. Previous research outlines the role of victimization, among a tapestry of other adverse childhood experiences in generating conditions conducive to heavy drinking behavior [48,71,72,73,74] often through a pathway leading from the traumatic experience to lasting or lagged psychological distress [27,41,49,51,75]. Our findings are strongly suggestive of childhood as a major life stage in which direct victimization has powerful and lasting results for women and girls, acting on the risk of heavy drinking, even controlling for adolescent victimization and more recent stressful events. For men and boys, however, the significance was a reduction in the risk of heavy drinking. Gender socialization is a possible explanation for this difference, as young men and boys are often socialized to externalize negative effect, potentially leading to higher levels of fighting or aggressive behavior (though not in all cases) [76,77] while young women and girls may be more likely to internalize coping through substance use [78].

Second, our findings contribute to the theoretical knowledge of heavy drinking behavior. While the theoretical perspectives guiding this study have differences, a core of stressful or straining events potentially leading to coping through substance use in the absence of social support or other protective factors provides opportunities for interpretation across health and criminological disciplines. As the self-medication hypothesis suggests, substance use behavior may be a feature of coping [28], and results from our study indicated traumatic events experienced in childhood among female respondents could lead to heavy drinking. Our findings partially substantiate the most important underlying theme from the self-medication hypothesis, that stress and distress can lead to coping through substance use [29]. The second theoretical framework, general strain theory, has broadened from its original focus to encompass related behaviors such as substance use [35,70,79,80]. As expected, our findings can be partially interpreted through this trenchant criminological lens. Our results indicated that direct victimization in childhood among female respondents was associated with heavy drinking, as suggested in the general strain theory. Heavy drinking is associated with a host of criminal-legal outcomes such as arrest and aggressive behavior/fighting [8,81] making it an important avenue for future programming within the criminal and juvenile justice system and public health alike.

The final theoretical framework, the stress-process model, has a deeper understanding of the stressors and symptomology associated with negative coping [42,82,83]. Again, our findings are partially in concordance with the core of the theory; direct victimization in childhood among women and recent stressful events among male respondents are both associated with heavy drinking. Depression was also associated with heavy drinking. The stress-process model also provides a better understanding through the examination of the role of depression and psychological distress as powerful mediators of risky health behavior [84]. We found depression to increase risk uniformly for both gender groups. A similar effect also reinforces the role of depression as a mediating variable within the general strain and stress-process frameworks [42,79,85]. Social support provides a safeguard against negative effect, and heavy drinking [82,86], and marriage was found to exert a protective effect for both gender groups. Social support did demonstrate an interactive effect with gender, with female respondents seeing greater reductions in risk for being married. Taken together, our findings illustrate the relevance of public health and criminological theory in discussing risky health behaviors that may also be illegal or associated with illegal activity.

Third, the interpolation of gender alongside other social statuses enriches our understanding of the nuanced social structure elements leading to or protecting against heavy drinking as an outcome [52]. Our findings suggest that female adolescents and young adults may face a greater risk of engaging in heavy drinking than their male counterparts in cases where victimization has occurred in childhood. Importantly, the interactive effect with gender indicates the childhood victimization’s effect is stronger (in generating heavy drinking risk) for female adolescents than for male adolescents for whom a decrease in risk was observed. Previous literature suggests that women’s experiences with sexual assault may be leading to differences in levels of psychological distress as well as later heavy drinking behavior [54,55]. Traumatic events early in the lifespan may forge a critical link in a chain of events leading to psychological distress (e.g., depression) to adult alcohol use or other substance use [51,87]. Heavy drinking also shares a stronger link to psychological distress and depressive symptoms in general [5], and specifically in older women compared to older men [59]. Notably, two other relevant gendered differences deserve mention as both are related to the protective factors against the literature, and socioeconomic measures provided key context. Parental education and possessing an associate’s or other college degree both exhibited an interactive effect with gender. Neither parental education nor possessing a college degree exerted any effect on male respondents’ propensity to engage in heavy drinking, while each decreased risk for female adolescents significantly. Higher levels of women entering and graduating colleges and universities in the United States may be increasing their level of capital to protect against recent stressors that we found associated with men’s heavy drinking in our study.

The picture becomes more complex when we interpolate race and gender, however. Our findings indicate that African American respondents are at significantly lower risk than their White counterparts, and Hispanic men are also at a significantly lower risk of heavy drinking. The interaction effect of race and gender is relevant, as the protective effect of race was weaker for Black males than Black females. The protective effect, however, is not found for Hispanic females, and again the results indicate an interactive effect among these two critical social status variables. The literature acknowledges African Americans generally have more risk factors for heavy drinking with lower rates of heavy drinking [62] than those who are White, but the risk is especially prominent for African American women [13]. Latino men and Latina women have also been found to have differential patterns and motivations (though psychological distress as a risk factor remains common among women) [88,89]. The literature and our results dovetail; gendered examinations of heavy drinking behavior remain relevant and are critical to engaging in meaningful prevention practices [8,52,53,87,90,91,92]. Future studies should make a careful study of social status modifying relationships between existing theoretical and thematic elements of heavy drinking.

Fourth, prevention efforts working at multiple levels must include modifying norms and beliefs about drinking behavior [93], including willingness and intention to engage in heavy drinking [3] and drinking contexts [14]. As well, research into emerging areas of neurobiology has led to better understandings of medical intervention models to reduce alcohol and other addictions in adulthood [94]. Heavy drinking prevention efforts in adolescents and young adults must acknowledge the social elements of drinking behavior that could be leading young adults through peer enticement or pressure to be successful [95]. The prevalence of cases in which depressive symptoms exist alongside heavy drinking points to the need for carefully constructed programming able to address the underlying traumatic experiences or other risk factors positioning these possibly endogenous outcomes [41,44]. Relatedly, trajectories for future programming require the careful inclusion of the intersection of sex, gender, sexual orientation, and gender identity, alongside but not instead of race [96,97,98]. Current programming may not yet be agile enough to address the specific needs of depressed women coping through heavy drinking [18]. Development of better programming remains important given these years of heavy drinking in adolescence, and early adulthood may lead to addictions in adulthood that are difficult to overcome without medical intervention [94]. Targeted prevention efforts, specifically addressing the adverse childhood experiences and other traumatic experiences leading to heavy drinking (and potentially greater negative outcomes) among women, remain a pressing need [62]. These must also meaningfully address how patterns of advantage and disadvantage structure the lives of women differently to address heightened risk among groups [13]. Previous research has demonstrated the effectiveness of brief motivational interventions or motivational interviews on drinking behavior, and these could be further refined to reach broader audiences, including adolescent and young women who are heavy drinking [17,18,63]. Another option that focuses on low-cost levels is text message or web-based programming aimed at reducing heavy drinking episodes or behaviors, which has demonstrated some effectiveness thus far in the literature for younger populations [54,99]. Promising approaches exist for treatment of heavy drinking behavior, and greater research and funding into both medical and other modalities would promote health equity in the United States and globally.

## 5. Conclusions

The current study extends the literature on victimization’s long-term consequences, with direct victimization in childhood exerting an effect on heavy drinking later in the lifespan among female respondents and recent stressful events associated with heavy drinking among male respondents. The results suggest that gender remains an important variable to examine in analyses of substance use and victimization-related sequelae. Specific differences among gender groups in our findings suggest early childhood adverse experiences are powerful predictors of later heavy drinking for girls and women, while they are also more protected by their own education and parental education against heavy drinking. This suggests an educational pipeline generating capital for women, but not men, in a way that protects them from heavy drinking. Race, socioeconomic status, and other important statuses, alongside gender, may play substantial roles in structuring heavy drinking outcomes; these should be investigated thoroughly in order to promote health equity among Americans. Our study demonstrated several strengths, primarily related to the multiple measures of victimization, occurring at different points in the lifespan, and these effects across time. We were further able to draw from both public health and criminology theory to better explain the roles of victimization, depression, and heavy drinking behavior and how it differs for male and female gender groups. We also unearthed several gender-specific differences in the risk and protective factors associated with heavy drinking, which can enrich the already-deployed programming, helping make it more relevant to community audiences.

Our study had a few limitations to note. First, our measures of victimization, both direct and indirect, are imperfect. Victimization, particularly vicarious or indirect victimization, is not a well-studied concept empirically, and large, nationally representative surveys have only a few indicators for use. The inclusion of additional indicators in these large surveys would help better elucidate these further. Next, while longitudinal, limitations in the data collection did not allow us a perfect year-for-year measurement of each indicator. While the gaps in collection years are small, additional years of data may paint a clearer picture of the link between victimization in childhood and later heavy drinking. Next, a host of negative affective states are relevant to discussions of criminal coping, including substance use. While depression has demonstrated some support, other affective states such as anxiety or anger may also be leading to heavy drinking behavior and are in need of substantial investigation to better plan successful programming that will reach broad audiences and reduce the harm of heavy drinking.

## Figures and Tables

**Table 1:** Descriptive statistics for time-varying heavy drinking outcome and independent variables.

	Whole Sample	Male	Female	*t*-Test	*χ2*
Variables	%/Mean (SD)	%/Mean (SD)	%/Mean (SD)	*p*	*p*
Heavy Drinking (0–1)	0.350	0.444	0.256		<0.01
Depression (5–20)	9.3 (2.4)	9.0 (2.3)	9.6 (2.4)	<0.01	
Stressful Events Index (0–6)	1.0 (0.9)	0.9 (0.9)	1.0 (0.9)	<0.01	
Age (19–36)	25.9 (4.0)	25.9 (4.1)	25.9 (4.0)	>0.05	
Completion of High School (0–1)	0.6150	0.6430	0.5870		<0.01
Completion of AA or Higher (0–1)	0.2940	0.2580	0.3300		<0.01
Income-to-Poverty Ratio (0–2627)	366.6 (380.0)	382.5 (390.4)	350.9 (368.7)	<0.01	
Married (0–1)	0.310	0.275	0.345	<0.01	
2004 Wave	0.195	0.193	0.197	>0.05	
2006 Wave	0.197	0.195	0.198	>0.05	
2008 Wave	0.204	0.203	0.207	>0.05	
2010 Wave	0.205	0.204	0.205	>0.05	
2015 Wave	0.200	0.201	0.198	>0.05	
*n* (person-waves)	22549	11210	11339		

Note: significance (*p*) of F-tests and significance of (*p*) of chi-square tests are presented on the right 2 columns.

**Table 2:** Results of generalized estimating equation (GEE) explaining heavy drinking for the whole sample.

	Model 1		Model 2	
Explanatory Variables	OR		OR	
Depression			1.0756	**
Black	0.4005	**	0.4051	**
Hispanic	0.8431	**	0.8579	**
Male	2.2405	**	2.3680	**
Age	0.9967		0.9960	
High School Diploma	1.0668		1.1053	
Associate/College Degree	1.2142	*	1.2723	**
Income-to-Poverty Ratio	1.0002	**	1.0002	**
Parent’s Education	1.0561	**	1.0553	**
Bully Victimization Younger Than 12	0.9796		0.9496	
Bully Victimization 12–18	1.0003		0.9657	
Seeing a Person Gunshot before 12	0.9910		0.9877	
Seeing a Person Gunshot 12–18	1.1323	*	1.1244	
Recent Stressful Events	1.0385	*	1.0300	*
Being Married	0.6441	**	0.6581	**
2006 Wave	1.0321		1.0406	
2008 Wave	0.9789		0.9719	
2010 Wave	0.8601		0.8715	
2015 Wave	0.77		0.78	
Constant	0	**	0.196771	**
Number of Person-Waves	22,549			
* *p* < 0.05; ** *p* < 0.01				

* indicates significant at the 0.05 level and ** indicates significant findings at the 0.01 level.

**Table 3:** Results of generalized estimated equations (GEE) explaining heavy drinking for each gender group among females versus males.

Explanatory Variables		Male				Female		
	Model 1		Model 2		Model 1		Model 2	
	OR		OR		OR		OR	
Depression			1.0742	**			1.0786	**
Black	0.4213	**	**0.4259**	**	0.3669	**	**0.3710**	**
Hispanic	0.8344	*	**0.8533**	*	0.8609		**0.8681**	
Age	1.0028		1.0033		0.9862		0.9842	
High School Diploma	1.0152		1.0378		1.1722		1.2463	
Associate/College Degree	1.1303		**1.1591**		1.3700	**	**1.4858**	**
Income-to-Poverty Ratio	1.0002	**	1.0002	**	1.0002	**	1.0002	**
Parent’s Education	1.0351		1.0323		1.0864	**	1.0881	**
Bully Victimization Younger than 12	0.8762	*	**0.8517**	*	1.2000	*	**1.1604**	*
Bully Victimization 12–18	0.9896		0.9480		1.0269		1.0001	
Seeing a Person Gunshot Before 12	0.9338		0.9343		1.0698		1.0548	
Seeing a Person Gunshot 12–18	1.0829		1.0785		1.2345		1.2079	
Recent Stressful Events	1.0528	*	1.0447	*	1.0212		1.0117	
Being Married	0.7880	**	**0.8036**	**	0.5025	**	**0.5149**	**
2006 Wave	1.0316		1.0359		1.0412		1.0536	
2008 Wave	0.9273		0.9155		1.0570		1.0552	
2010 Wave	0.8049		0.8101		0.9487		0.9676	
2015 Wave	0.6967		0.6991		0.8830		0.9161	
Constant	0.8795		0.4502		0.4392		0.2054	**
Wald Chi-Square	282.26	**	342.20	**	388.25	**	437.79	**
Number of Person-Waves	11,210		11,210		11,339		11,339	
* *p* < 0.05; ** *p* < 0.01								

Note: Bold-faced, underlined figures signify significant interaction effects involving gender and the independent variable. * indicates significant at the 0.05 level and ** indicates significant findings at the 0.01 level.

## Data Availability

Data from this study are publicly available and accessible through the United States Bureau of Labor Statistics. https://www.bls.gov/nls/nlsy97.htm (accessed on 11 October 2021).
